# Geographic Variation in Diet and Genetic Connectivity of Populations of the Widespread Predatory Cone Snail *Conus miliaris*

**DOI:** 10.1002/ece3.72434

**Published:** 2025-11-04

**Authors:** Thomas F. Duda, Diana Carolina Vergara‐Flórez

**Affiliations:** ^1^ Department of Ecology and Evolutionary Biology and Museum of Zoology University of Michigan Ann Arbor Michigan USA

**Keywords:** feeding ecology, geography, population structure, predator–prey interactions

## Abstract

Knowledge of how traits of widely distributed species vary across geographic scales is important for understanding patterns of intraspecific variation and the source of this variation. *Conus miliaris* is a broadly distributed predatory gastropod that exhibits tremendous differences in feeding ecology among populations separated by thousands of kilometers (i.e., at Guam, American Samoa, and Rapa Nui). Here, we evaluated patterns of variation of diets of populations of *C. miliaris* from sites in French Polynesia to determine how diets differ over smaller areas through examination of mitochondrial 16S sequences from fecal samples. To determine how patterns of variation in feeding ecology are associated with population structure and connectivity, we examined sequences of a mitochondrial gene region (i.e., *COI*) and two nuclear (i.e., conotoxin) loci. Populations in French Polynesia show overlapping and relatively broad diets, but prey items and dietary breadth of these populations tend to be more similar to those in Rapa Nui than in Guam and American Samoa. While examination of *COI* sequences shows little to no structure among populations from Guam, American Samoa, and French Polynesia and strong divergence of these populations from the population in Rapa Nui, analyses of conotoxin loci reveal some admixture between French Polynesia and Rapa Nui populations. These results suggest that the broad dietary breadth and similarity in diets of populations from French Polynesia and Rapa Nui reflect the origin of this ecological phenotype in Rapa Nui and the subsequent migration of it into French Polynesia.

## Graphical abstract

We examined patterns of dietary and genetic variation of a broadly distributed, Indo‐West Pacific cone snail at four sites in French Polynesia and compared them to populations at Guam, American Samoa, and Rapa Nui. Our results indicate that populations at French Polynesia possess broad and overlapping diets that are most similar to those of Rapa Nui. Population genetic results further reveal admixture between Rapa Nui and French Polynesia which suggests that the broad diet originated in Rapa Nui and extended into French Polynesia via dispersal from Rapa Nui.

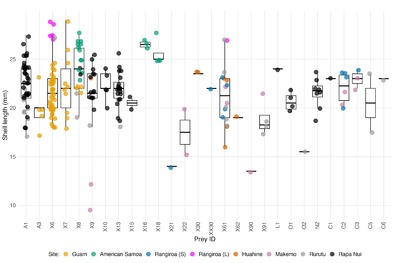

## 1 Introduction

Populations of broadly distributed species are often exposed to a variety or gradient of environmental conditions at sites throughout their ranges which may cause differentiation of traits associated with the different settings (Hargreaves 2024; Moran et al. 2016; Wadgymar et al. 2022). While information from geographically distant populations can reveal how traits differ broadly, knowledge of how they vary at smaller spatial scales is important for understanding the geographic scale and patterns at which these traits vary and whether they may be due to phenotypic plasticity or genetic variation (Moran et al. 2016).

The marine gastropod family Conidae (i.e., “cone snails”) has diversified tremendously since its origin in the Miocene, approximately 55 mya (Kohn 1990). The group contains 1062 recognized extant species (Ahyong et al. 2024). Species are distributed throughout the world's oceans and largely occur in shallow tropical areas although some occur at deeper depths and in subtropical and temperate zones (Röckel et al. 1995). Like other members of the superfamily Conoidea, cone snails are predatory snails that utilize venom to capture prey. Cone snails also exhibit tremendous interspecific differences in diet and a few broadly distributed cone snail species have been found to exhibit geographic variation in diet (Chang et al. 2015; Duda and Lee 2009a; Kohn 1978; Weese and Duda 2019).

**FIGURE 1:**
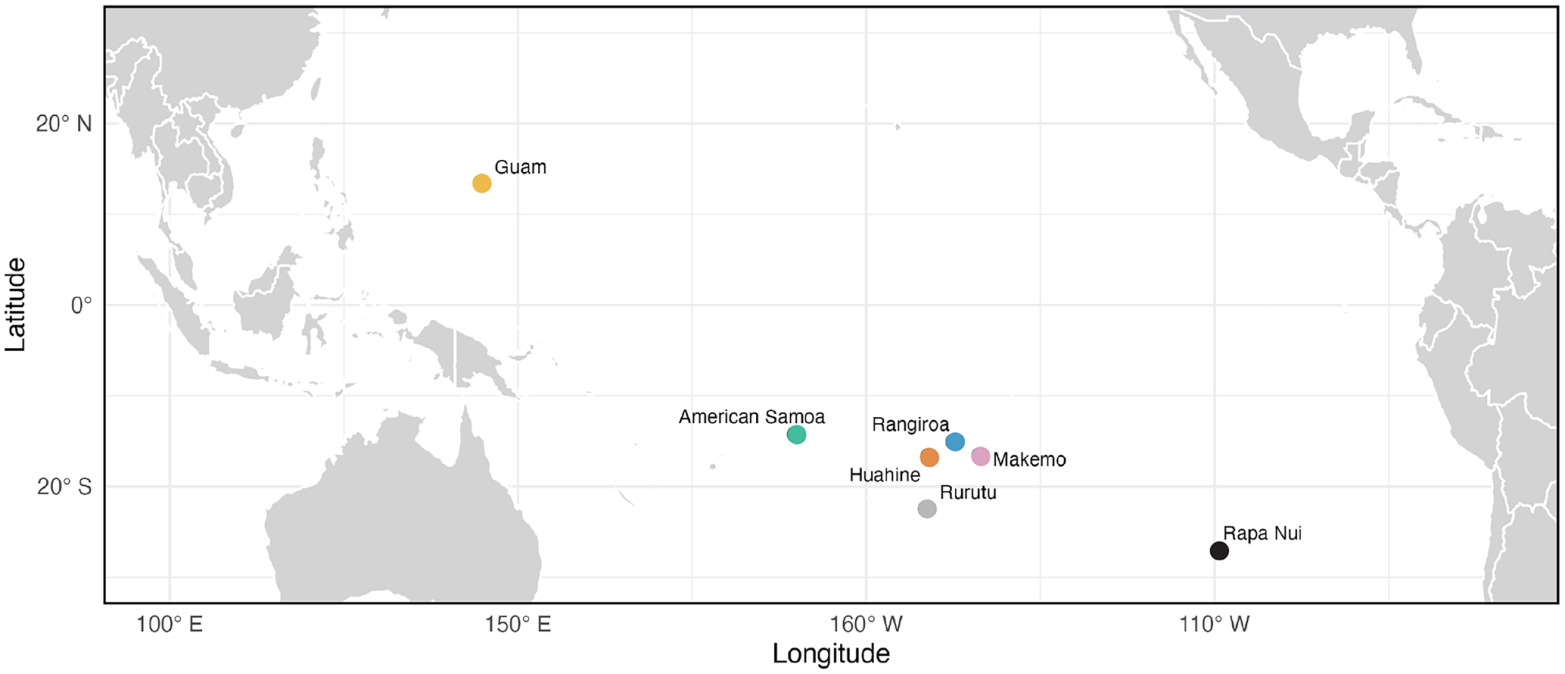
Map of the Pacific showing locations of sites. *Conus miliaris* occurs throughout shallow water regions of the Indo‐West Pacific except at the Hawaiian Archipelago and Marquesas Islands (Röckel et al. 1995).

*Conus miliaris* is an annelid‐eating cone snail species that occurs throughout most shallow, tropical, and subtropical areas of the Indo‐West Pacific region (Röckel et al. 1995). It is the only cone snail species with an established population at Rapa Nui (Kohn 1978), which is located at the southeastern edge of this vast region (see Figure 1). *C. miliaris* appears to have undergone ecological release at Rapa Nui, where it exhibits a much larger dietary breadth compared to populations elsewhere presumably due to the absence of congeners at Rapa Nui (Kohn 1978). Indeed, while populations at other sites that co‐occur with other cone snail species tend to only prey on eunicid annelids, prey items at Rapa Nui also include members of other annelid families, including Capitellidae, Lumbrineridae, Nereididae, and Onuphidae (Kohn 1978; Weese and Duda 2019).

Based on analyses of DNA sequences obtained from feces of *C. miliaris* at Guam, American Samoa, and Rapa Nui, this species exhibits very little overlap in prey utilization among these populations (Weese and Duda 2019). These three sites, however, are separated by thousands of kilometers (Figure 1) and given the large distances between them may hold distinct assemblages of prey items or access to prey may be affected by differences in the presence or absence of competitors or environmental conditions. How diets differ over smaller geographic scales within more constrained regions, however, is not currently well understood. In *the* seminal paper on the ecology of cone snails, Kohn (1959) reports that prey utilization patterns of some cone snail species differ depending on habitat type and conditions at sites in the Hawaiian Archipelago. For example, diets of *Conus abbreviatus* and *Conus ebraeus*, two other vermivorous cone snails, differ depending on whether they occur on a marine bench or subtidal reef habitat and if algal mats are particularly abundant (Kohn 1959). Hence, although Kohn showed that populations of potential prey and diets differ depending on habitat conditions, we do not yet know if populations from relatively closely distributed sites exhibit similar prey utilization patterns or if they show the same low levels of overlap in diet as observed for populations from distant archipelagos. Here we identify prey items of *C. miliaris* at four sites in French Polynesia (see Figure 1) to determine if and how diets differ at a finer geographic scale than examined previously. Prey items at sites in French Polynesia may overlap with each other (and possibly with those observed previously at American Samoa given the relative close proximity of French Polynesia to American Samoa, see Figure 1) if their diets reflect regional similarity in the availability of potential prey items. Moreover, we expect that populations at sites in French Polynesia exhibit narrow dietary breadths given that several congeners (e.g., *Conus chaldaeus*, *Conus coronatus, Conus ebraeus*, *Conus flavidus*, *Conus frigidus*, *Conus lividus*, *Conus miles*, *Conus nanus*, *Conus rattus*, *Conus sanguinolentus*, and *Conus sponsalis* ) typically occur at these sites (Richard and Rabiller 2021).

To evaluate patterns of variation in prey utilization of *C. miliaris* at sites in French Polynesia and compare diets of these populations to previously determined diets from populations at Guam, American Samoa, and Rapa Nui, we obtained 16S sequences from fecal materials from four locations representing three archipelagos in this region: Huahine (Society Islands), Rangiroa and Makemo (Tuamotu Archipelago), and Rurutu (Austral Islands). We used the sequences obtained from feces to infer putative prey species based on relationships of sequences in gene tree reconstructions that include sequences from prior studies and annelid sequences from GenBank. We estimated levels of dietary overlap by comparing the relative frequencies of prey items observed at different sites and calculated dietary breadth statistics based on prey frequencies and their sequences. To determine if ontogenetic shifts in diet and differences in size frequency distributions of individuals sampled from different sites may be contributing to low levels of dietary overlap, we compared size frequency distributions of sites and evaluated whether or not prey utilization patterns are associated with the sizes of individuals.

Furthermore, *C. miliaris* shows evidence of population genetic structure in the Indo‐West Pacific (Duda and Lee 2009a, 2009b; Weese and Duda 2019). In particular, the population at Rapa Nui is genetically isolated from populations at Guam and American Samoa based on analyses of sequences of a region of the mitochondrial cytochrome oxidase subunit I gene (*COI*) (Duda and Lee 2009b) and two venom‐associated genes (O‐superfamily conotoxin loci) (Duda and Lee 2009a) as well as patterns of variation of single nucleotide polymorphisms at venom and non‐venom related transcripts as revealed through comparison of venom duct transcriptomes (Weese and Duda 2019). Moreover, although populations at Guam and American Samoa do not exhibit differentiation at the two O‐superfamily loci (Duda and Lee 2009a), venom‐associated transcripts show higher levels of divergence than non‐venom transcripts among all three populations which suggests that venom genes are under selection possibly due to the differences in diet among these populations (Weese and Duda 2019). Because Rapa Nui is so geographically isolated from other sites in the Indo‐West Pacific and given the relatively close proximity and presence of islands with suitable habitats for *C. miliaris* between American Samoa and French Polynesia, we anticipate that populations at French Polynesia are more genetically allied with populations at Guam and American Samoa than with the population at Rapa Nui. To test this hypothesis, we obtained sequences of *COI* and the two O‐superfamily loci from individuals from sites at French Polynesia and compared them to sequences obtained previously from Guam, American Samoa, and Rapa Nui.

## 2 Materials and Methods

### 2.1 Samples and Collection Sites

We collected individuals of *Conus miliaris* at sites on the seaward side (14°56′54″ S 147°40′28″ W) and within the lagoon (14°57′13″ S 147°40′17″ W) at Rangiroa, along the seaward side of Makemo (16°33′10″ S 143°44′23″ W, 16°36′31″ S 143°37′31″ W, 16°36′54″ S 143°36′44″ W, 16°37′19″ S 143°35′49″ W, and 16°37′11″ S 143°34′03″ W) (we did not detect individuals of this species within the lagoon), Huahine (16°42′27″ S 151°02′31″ W), and Rurutu (22°26′21″ S 151°22′32″ W) (coordinates determined from Google Earth) (Figure 1). The seaward sites at atolls of Rangiroa and Makemo are similar in that they are marine bench habitats, although macroalgae were particularly abundant at Rangiroa (as they were at other sites) while they were largely absent at Makemo. The lagoon site at Rangiroa represents a subtidal reef. Collections at Huahine and Rurutu took place in a relatively shallow back reef area.

We measured shell sizes (shell height and width and height to shoulder) to the nearest 0.5 mm with modified (“by rhinoplasty” (Kohn and Riggs 1975)) vernier calipers. Because some individuals were heavily encrusted with coralline algae, we were not able to obtain measurements from all specimens. We placed specimens in separate small containers with seawater and then transferred suspected fecal materials into cryovials with a pipette, removed as much seawater as possible, and filled tubes with 95% ethanol. We kept specimens in containers for up to five days and except for five voucher specimens from each site (which were preserved in 95% ethanol) returned them to their original site of collection after obtaining a small sample of foot tissue (which was also preserved in 95% ethanol) from a set of individuals from each site.

### 2.2 Fecal Analyses

We examined suspected fecal materials under a compound microscope to determine if annelid setae were present and obtain a tentative identification of prey taxa. We transferred a small piece of fecal material to a centrifuge tube containing 350 μL of lysis buffer (from the EZNA Mollusk DNA extraction kit, Omega Bio‐tek, Norcross, USA). We extracted DNA following slight modifications to the kit's suggested protocol (i.e., we did not include an RNase digestion step). We performed amplifications with 1 μL of the DNA extraction, final concentrations of 0.5 μM of annelid‐specific primers (forward primer 16SANNf2 and reverse primer 16Spr1 (Duda et al. 2009)) (primers were each appended with one of two vector primers (M13 Forward and Reverse) for sequencing with these vector primers), and 5 μL of the 2X GoTaq Green Master Mix (Promega Corp, USA) at 10 μL final volumes. The amplification consisted of 40 cycles with a 94°C and 30 s denaturation step, 57°C and 30 s annealing step, and a 72°C and 30 s extension step. The primers amplify approximately a 340 to 350 bp region of the mitochondrial 16S rRNA gene. We ran amplification products in a 1.5% agarose/0.5X TBE gel in 0.5X TBE running buffer for 12 min at 100 V. We prepared templates for sequencing by diluting them 1:5 in water and then submitted them to GENEWIZ (Azenta Life Sciences, South Plainfield, NJ, USA) or Eurofins Genomics LLC (Louisville, KY, USA) for sequencing. We examined chromatograms and edited sequences in Geneious Prime v2024.0.7 (Dotmatics, Woburn, MA, USA) and exported sequences for subsequent analyses.

### 2.3 Dietary Analyses

We created an alignment of new prey sequences and previously published prey sequences of *C. miliaris* from Guam, American Samoa, and Rapa Nui (GenBank accession numbers MH634087‐MH634256) using Seqotron v1.01 (Fourment and Holmes 2016). To evaluate taxonomic identities of prey taxa, we used BLAST to identify sequences from annelids that are similar to all unique prey sequences in the alignment. We downloaded best matches of sequences from GenBank (based on lowest E value scores and percent identities), aligned them with our prey sequences, and created separate alignments of disparate taxa (i.e., capitellids, eunicids, and nereids). We built gene trees with maximum likelihood approaches in Seqotron to evaluate relationships of GenBank annelid sequences and our prey sequences. We then retained all GenBank sequences that clustered most closely to the prey sequences and removed others to create final alignments. We constructed neighbor‐joining (Saito and Nei 1987) trees for each final alignment using Kimura 2‐parameter (Kimura 1980) distances and pairwise deletion of gaps or missing data with 500 bootstrap replicates (Felsenstein 1985) using MEGA11 (Stecher et al. 2020; Tamura et al. 2021). We examined gene trees and either assigned previously designated prey taxon codes (from Weese and Duda (2019)) to sequences that cluster closely with previously published prey sequences or assigned new codes for putative prey taxa that were not observed in prior work. Prey codes designate a unique sequence or set of sequences that group tightly together in gene trees and show few base substitutions among them such that each prey code putatively represents a different prey species.

We compared diets of populations based on the observed frequencies of prey items by calculating proportional similarity indices (PS_I_ values, Whittaker 1952). We performed 1000 permutations to determine if the values calculated from observed data are less than 95% of the values estimated from the permutated data. Permutations maintained both the original sample sizes of each population and the total number of observations for each putative prey species. We calculated summary statistics for each population (i.e., number of prey sequences, number of prey species, and Shannon (1948) diversity indices).

To determine the phylogenetic disparity of prey taxa of each population based on average genetic distances among sequences of each population, we first created separate alignments of prey sequences for each population. Because sequences of the same prey item differed in length owing to differences in the quality of the recovered chromatograms, we used the longest sequence that represents each prey taxon in these alignments. We then calculated the average Kimura 2‐parameter (Kimura 1980) distances with pairwise deletion of gaps or missing data and standard errors (based on 500 bootstrap replicates) for each population with MEGA11 (Stecher et al. 2020; Tamura et al. 2021).

To determine if prey utilization patterns are associated with sizes of individuals, we first compared size frequency distributions (based on shell lengths) among sites using Kruskal‐Wallis and pairwise Wilcoxon tests. We also performed these tests to determine if there is a significant association between prey items and sizes of individuals (i.e., shell lengths) that consume them. We performed these tests for each site and pooled individuals from all sites. We corrected for multiple tests using the Benjamini‐Hochberg (Benjamini and Hochberg 1995) method. We also constructed plots of sizes of individuals that consumed different prey taxa at each site and combined sites to illustrate the relationship between prey utilization patterns and sizes of individuals.

### 2.4 DNA Sequences From Populations

We extracted DNA from approximately 5 mg of foot tissue of 20–30 individuals from Huahine, Rangiroa, Rurutu, and Makemo using the E.Z.N.A. Mollusk DNA Kit as described above. We amplified a 658 bp region of the mitochondrial cytochrome oxidase subunit I (*COI*) gene with Folmer primers LCO1490 and HCO2198 (Folmer et al. 1994) that were each appended with one of two vector primers (M13 Forward and Reverse) for sequencing with these vector primers. We amplified sequences of a 132 bp region of two O‐superfamily conotoxin loci (*MIL2* and *MIL3*) using locus‐specific forward primers (MIL2C and MIL3E, respectively (Duda and Lee 2009a)) and a general O‐superfamily conotoxin gene reverse primer (TOX2 (Duda and Palumbi 1999)); primers were also appended with vector primers as described above. We performed 40 cycles of a 94°C/30 s denaturation step, 30 s annealing step with a 45°C annealing temperature for *COI* amplifications and a 57°C annealing temperature for *MIL2* and *MIL3* amplifications, and a 72°C/30 s extension step. We visualized and prepared amplification products as described above and submitted them for sequencing to Eurofins Genomics LLC (Louisville, KY, USA). We analyzed resultant chromatograms with Geneious Prime. For sequences of MIL2 and MIL3, we inferred alleles of chromatograms with overlapping peaks (representing putative heterozygous conditions) by comparing the sequences inferred from the chromatograms to previously described alleles (Duda and Lee 2009a).

### 2.5 Population Genetic Analyses

We aligned *COI*, *MIL2*, and *MIL3* sequences with those of individuals of *C. miliaris* from Guam, American Samoa, and Rapa Nui as analyzed by Duda and Lee (2009a, 2009b) (GenBank accession numbers FJ392914‐FJ392994, FJ411486‐FJ411515, FJ613506‐FJ613520, and FJ716816‐FJ716827). We estimated F‐statistics (i.e., pairwise F_ST_ values) and significance values using Arlequin v3.5.2.2 (Excoffier and Lischer 2010) based on haplotype frequencies of the partial *COI* sequences and allele frequencies of *MIL2* and *MIL3*.

For analyses of population structure using genotype data for nuclear loci *MIL2* and *MIL3*, we excluded individuals with missing genotype calls for one of the two loci. We utilized Structure v2.3 (Pritchard et al. 2000) to evaluate support for different values of *K* (i.e., the number of inferred population clusters) using models with and without admixture, burnin of 100,000 iterations, and a total run length of 1,000,000 iterations. We performed 10 separate runs for each *K* value (i.e., 1 through 7) and then used Structure Harvester (Earl and vonHoldt 2012) to compile and parse the Structure results and apply the Evanno et al. method (2005) for estimating support for different *K* values based on the deltaK statistic (i.e., the rate of change between log likelihood values estimated for adjacent *K* values). We also used rmavericK v1.1.0 (Verity, n.d.) in R (R Core Team 2024) to further evaluate support for different *K* values using models with and without admixture, 50 rungs (i.e., MCMC chains), a minimum burnin length of 2000 iterations (which covered the minimum number of iterations to reach convergence for all values of *K*), and a total run length of 10,000 iterations while ensuring occurrence of non‐zero coupling acceptance rates (i.e., rates at which information is successfully passed between chains). The approach used by rmaverick for determining support for different *K* values is distinct from that used by the Evanno et al. (2005) deltaK approach in that it utilizes “thermodynamic integration” to evaluate support for different models (Verity and Nichols 2016) and therefore provides an alternative means to evaluate which *K* value best explains the observed data. We plotted posterior assignments to clusters; we used average assignments from replicate runs from structure that were calculated from the output from Structure Harvester.

## 3 Results

### 3.1 Dietary Analyses

**FIGURE 2:**
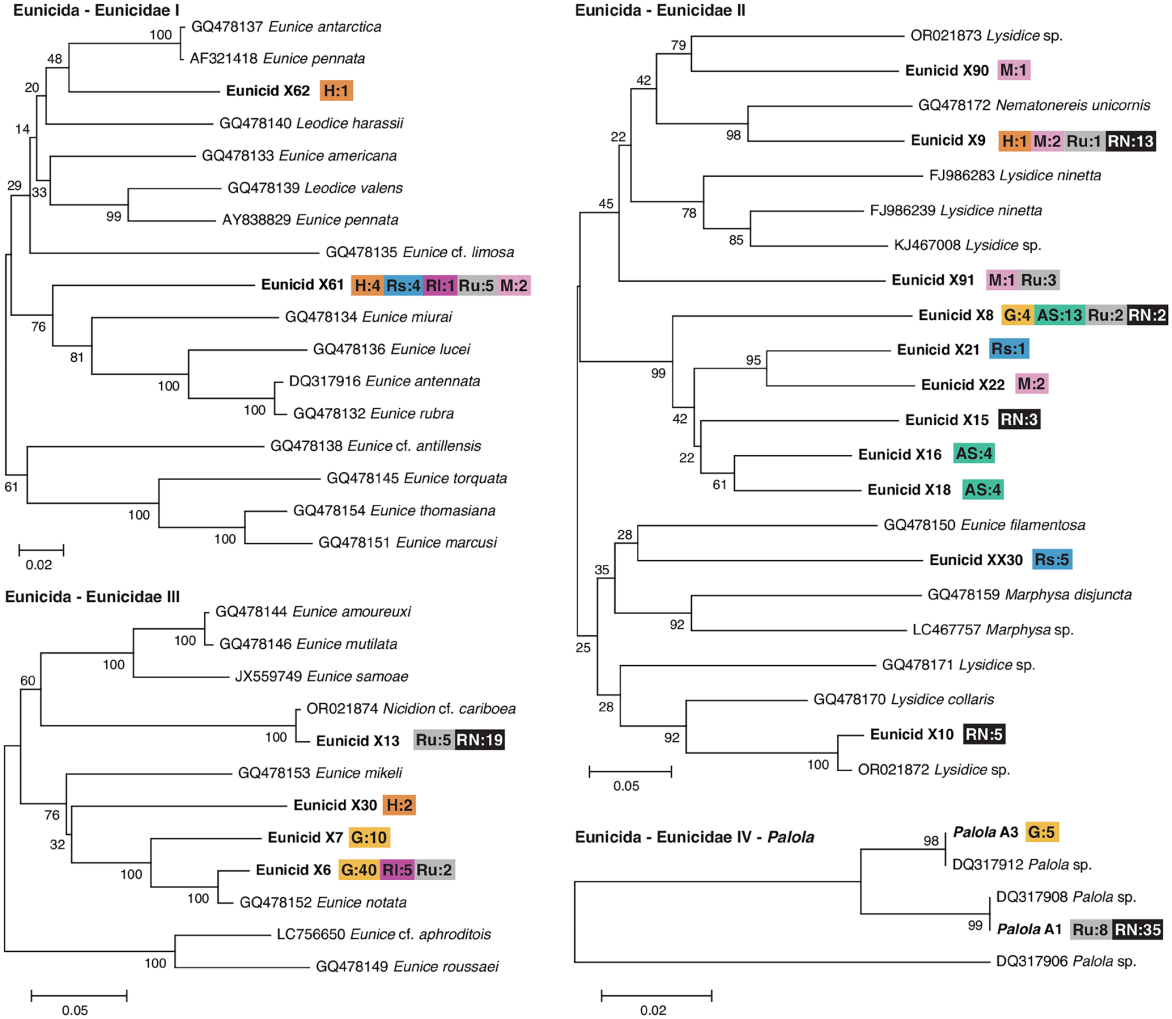
Gene trees of 16S sequences obtained from feces of *C. miliaris* at French Polynesia; previously published sequences of prey of *C. miliaris* from Guam, American Samoa, and Rapa Nui; and sequences of annelids from GenBank. Eunicidae I–IV. Names of sequences of prey items in bold typeface; those from GenBank begin with the accession number and include the taxon name that was submitted with the sequence. The number of prey sequences obtained from each site is indicated with site abbreviations: AS, American Samoa; G, Guam; H, Huahine; M, Makemo; Rl, Rangiroa (lagoon); RN, Rapa Nui; Rs, Rangiroa (seaward); Ru, Rurutu. Trees midpoint rooted; bootstrap values indicated on branches.

**FIGURE 3:**
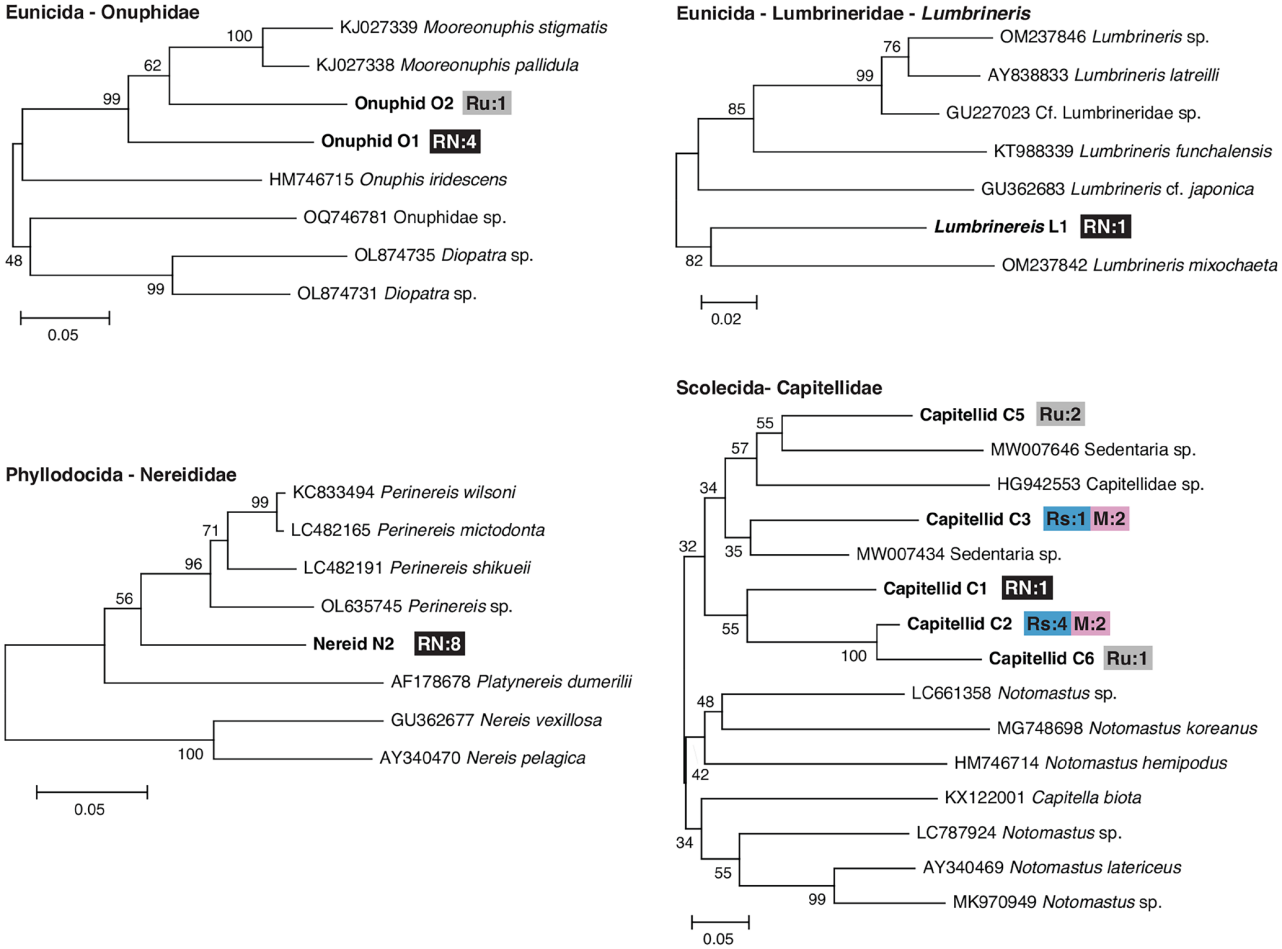
Gene trees of 16S sequences obtained from feces of *C. miliaris* in French Polynesia; previously published sequences of prey of *C. miliaris* from Guam, American Samoa, and Rapa Nui; and sequences of annelids from GenBank. Onuphidae, Lumbrineridae, Nereididae, and Capitellidae. Names of sequences and other items as in Figure 2.

We obtained sequences of prey items from 8 individuals of *C. miliaris* from Huahine, 11 from the seaward side and 6 from the lagoon at Rangiroa, 26 from Rurutu, and 12 from Makemo (Table 1) (GenBank accession numbers PV540063‐PV540125). Based on the gene tree constructed with these sequences, previously published prey sequences of *C. miliaris* , and annelid sequences that are the best matches to the prey sequences, only three prey items cluster tightly with sequences from GenBank including eunicid X13 (*Nicidion* cf. *cariboea*, accession number OR021874), eunicid X6 ( *Eunice notata* , GQ478152), eunicid X10 (*Lysidice* sp., OR021872), and *Palola* A1 and A3 (*Palola* sp., DQ317908 and DQ317912, respectively, which are members of Shulze's (2006) clades “A1” and “A3”) (Figures 2 and 3). The remaining sequences do not show high similarity with sequences in GenBank and so the taxa that they represent can only be ascertained to the family level.

**TABLE 1:** Summary statistics for dietary characterizations from each site with the number of individuals from which prey sequences were recovered (N), average shell length of individuals and standard deviations (SD), number of prey items (i.e., inferred species), Shannon diversity indices (*H*′), and average Kimura 2‐parameter genetic distances among recovered prey sequences (genetic disparity) with standard errors (SE) based on 500 bootstrap replicates.

Site	*N*	Average shell length, mm (SD)	*N*, prey items	*H*′	Genetic disparity (SE)
Guam	59	21.3 (2.18)	4	0.96	0.124 (0.012)
American Samoa	21	25.7 (2.32)	3	0.93	0.153 (0.015)
Huahine	8	21.1 (2.99)	4	1.21	0.295 (0.024)
Rangiroa, seaward	11	21.3 (2.94)	5	1.40	0.470 (0.039)
Rangiroa, lagoon	6	27.8 (0.84)	2	0.45	0.128 (0.016)
Rurutu	26	20.1 (2.90)	10	2.02	0.410 (0.027)
Makemo	12	18.4 (4.61)	7	1.91	0.524 (0.038)
Rapa Nui	90	22.4 (1.91)	10	1.76	0.323 (0.025)

Individuals from sites at French Polynesia share five of the 16 prey species that were previously determined from sequences of feces of individuals at Guam, American Samoa, and Rapa Nui (i.e., eunicids X6, X8a, X9, and X13 and *Palola* A1), while 13 other prey items are unique to individuals from French Polynesia (i.e., eunicids X21, X22, X30, X61, X62, X90, X91, and XX30; onuphid O2; and capitellids C2, C3, C5, and C6) (Figures 2 and 3). Within French Polynesia, we detected six prey items from more than one site. These include eunicid X61 that was observed at all sites, eunicid X6 at the lagoon at Rangiroa and at Rurutu; eunicid X91 at Makemo and Rurutu; X9 at Huahine, Makemo, and Rurutu; capitellid C2 and C3 at the seaward site at Rangiroa and at Makemo (Figures 2 and 3). Other prey items are unique to single sites, including eunicid X30 and X62 at Huahine, eunicid X22 and X90 at Makemo, eunicid XX30 at the seaward site at Rangiroa, and onuphid O2 and capitellids C5 and C6 at Rurutu (Figures 2 and 3). Several prey items remain unique to other sites outside of French Polynesia, including eunicid X7 and *Palola* A3 at Guam; eunicids X16 and X18 at American Samoa; and eunicids X10 and X15, onuphid O1, lumbrinerid L1, nereid N2, and capitellid C1 at Rapa Nui (Figures 2 and 3).

Sites in French Polynesia show variation in the diversity of the prey items they utilize with Shannon diversity indices and average genetic distances among prey sequences ranging from 0.45 to 0.128, respectively, for the lagoon at site at Rangiroa (i.e., values lower than those calculated for Guam and American Samoa), to much larger values for sites at Rurutu (2.02 and 0.410) and Makemo (1.91 and 0.524) (i.e., values that are greater than those observed at Rapa Nui (1.76 and 0.323) despite a much smaller sample size at these sites) (Table 1). Furthermore, while the Shannon diversity index at the seaward site at Rangiroa (1.40) is not much larger than values observed at Guam (0.96) and American Samoa (0.93), the average genetic distance among its prey sequences (which serves as a better proxy for the phylogenetic disparity of its prey) is greater than the value estimated for Rapa Nui (0.323) (Table 1). In addition, although Rapa Nui is the only site at which *C. miliaris* was previously known to prey on taxa outside of Eunicidae, including a lumbrinerid and nereid that have not been identified as prey items at the other sites, individuals at Rurutu, the seaward site at Rangiroa, and Makemo also prey upon non‐eunicid annelids (i.e., capitellids and/or onuphids), while only eunicids were observed as prey items at other sites, including Huahine and the lagoon site at Rangiroa as well as Guam and American Samoa (Figure 2).

**FIGURE 4:**
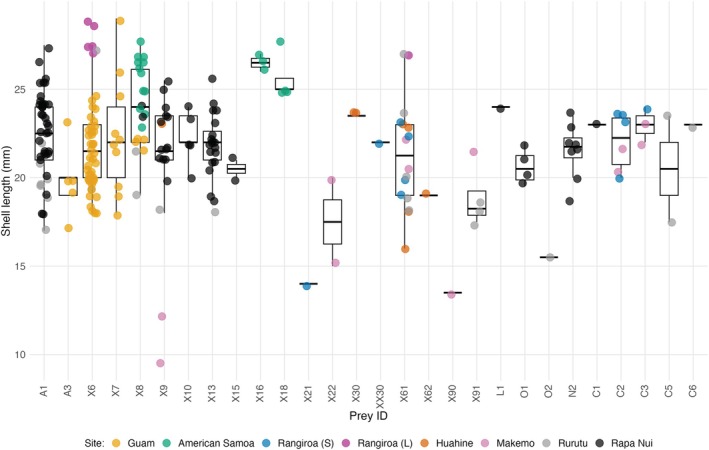
Box plots of shell lengths of individuals utilizing different prey items with colors indicating the sites examined. Legend shows colors for different sites; Rangiroa (S) = seaward site at Rangiroa, Rangiroa (L) = lagoon site at Rangiroa.

Diets show considerable overlap among sites at French Polynesia with PS_I_ values ranging between 0.167 and 0.364 and *p*‐values at or above 0.147 (Table 2). All but one site in French Polynesia shows significantly nonoverlapping diets between sites at American Samoa and Guam; the exception is the lagoon at Rangiroa site in which eunicid X6 comprises the majority of the prey sequences detected at this site and at Guam (Table 2, Figure 2). While frequencies of prey items utilized at seaward and lagoon sites of Rangiroa are significantly different from those at Rapa Nui, diets at Rapa Nui and Huahine, Makemo, and Rurutu overlap with PS_I_ values ranging between 0.125 and 0.407 and associated *p* values between 0.08 and 0.536 (Table 2). The boxplot of shell sizes of individuals reveals patterns of prey utilization by individuals of different sizes and sites (Figure 4).

**TABLE 2:** Measures of dietary overlap among populations of *C. miliaris* at sites in the Pacific.

	Guam	American Samoa	Huahine	Rangiroa (S)	Rangiroa (L)	Rurutu	Makemo	Rapa Nui
Guam		**0.002**	**0.002**	**0.002**	0.857	**0.026**	**0.001**	**< 0.001**
American Samoa	**0.068**		**0.006**	**0.001**	**0.008**	**0.002**	**0.002**	**< 0.001**
Huahine	**0.000**	**0.000**		0.481	0.189	0.319	0.441	0.083
Rangiroa (S)	**0.000**	**0.000**	0.364		0.147	0.166	0.672	**< 0.001**
Rangiroa (L)	0.678	**0.000**	0.167	0.167		0.347	0.212	**< 0.001**
Rurutu	**0.145**	**0.077**	0.231	0.192	0.244		0.422	0.539
Makemo	**0.000**	**0.000**	0.292	0.424	0.167	0.288		0.075
Rapa Nui	**0.022**	**0.022**	0.125	**0.000**	**0.000**	0.407	0.144	

*Note:* Proportional similarity indices (PS_I_ values) for pairwise comparisons of frequencies of prey items observed at different sites (below diagonal) and *p* values estimated from 1000 permutations (above diagonal). *p* values less than 0.05 and their corresponding PS_I_ values are in bold typeface.

**FIGURE 5:**
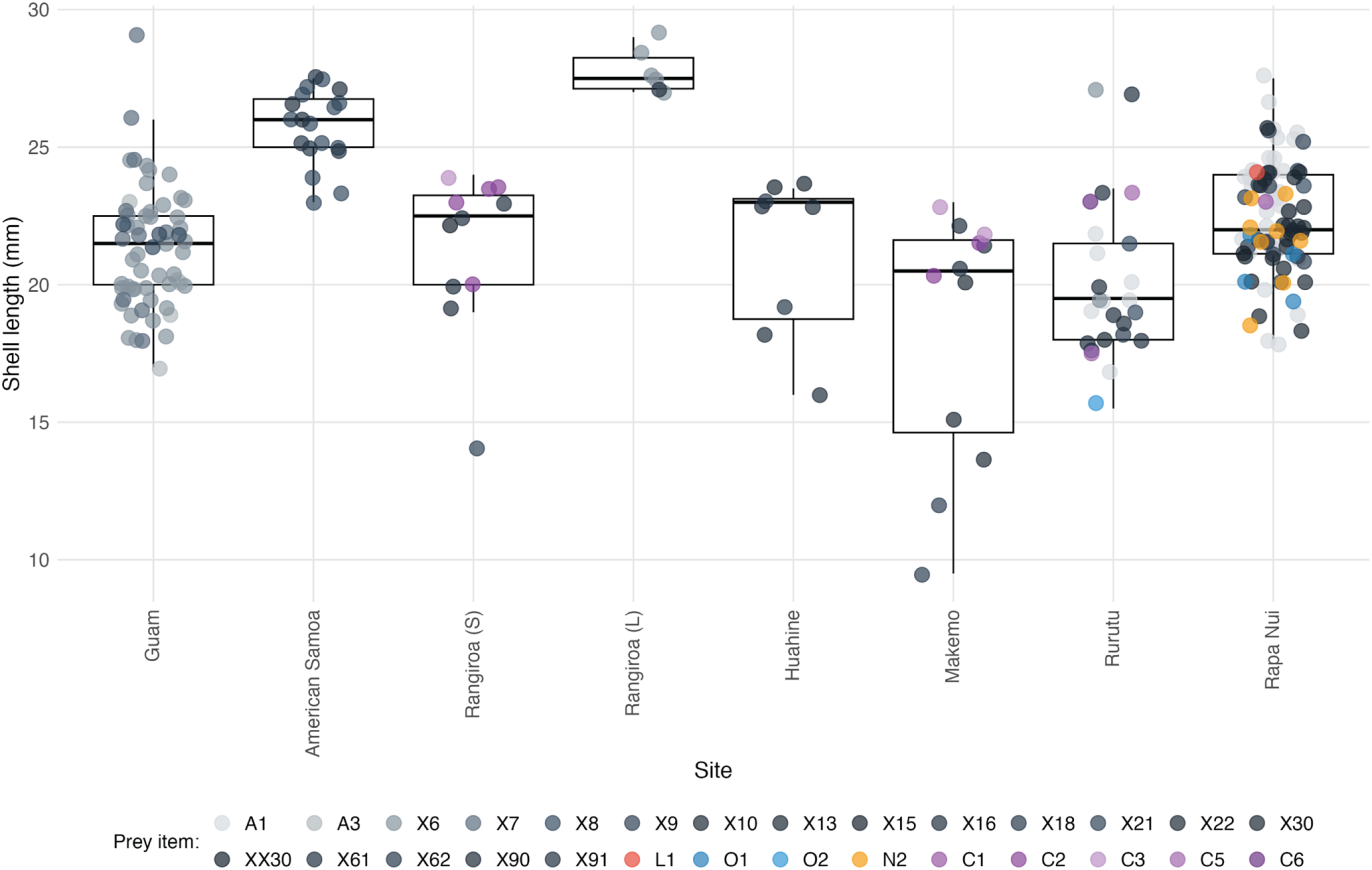
Box plots of shell lengths of individuals from each site with colors indicating the prey item utilized. Legend shows colors for different prey items with colors in different shades of gray for eunicids (i.e., A1‐X91) and other colors for other annelid families (i.e., L1‐C6) (see Figures 2 and 3 for families). Rangiroa (S) = seaward site at Rangiroa, Rangiroa (L) = lagoon site at Rangiroa.

**FIGURE A1:**
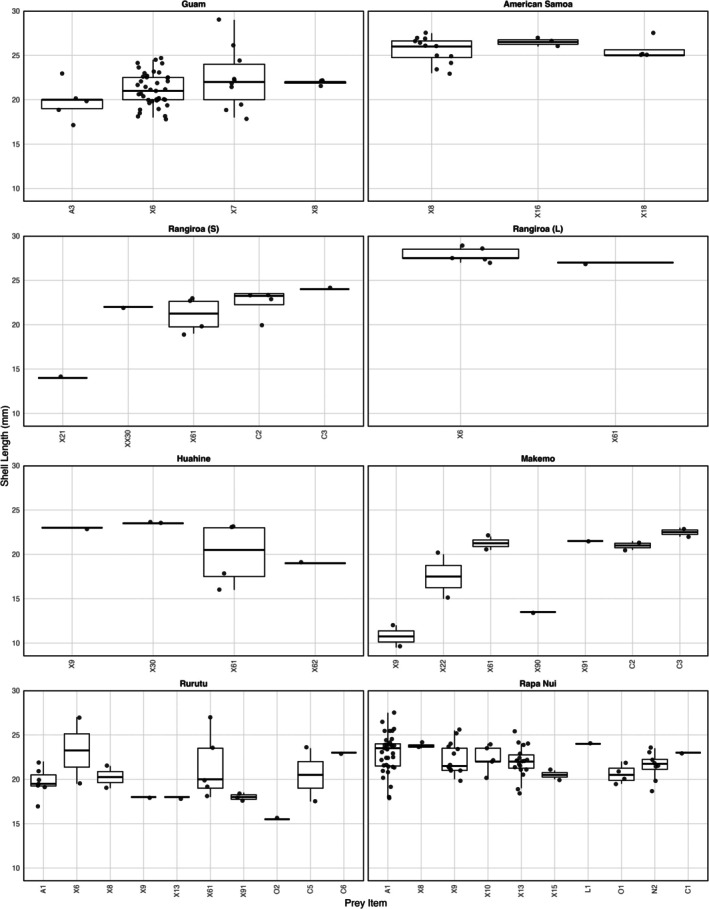
Box plots of shell lengths of individuals from each site for prey items. Rangiroa (S) = seaward site at Rangiroa, Rangiroa (L) = lagoon site at Rangiroa.

Size frequency distributions differ significantly among sites (*p* value = 2.8 × 10^−14^). Based on results from Wilcoxon tests, the comparisons that contribute to this result are all of those involving American Samoa and the lagoon site at Rangiroa and the comparisons for Guam–Rurutu, Guam–Rapa Nui, Rurutu–Rapa Nui, and Makemo–Rapa Nui (see Figures 4 and 5). When examining information from all sites, sizes of individuals that consume different prey also differ significantly (*p* value = 4.4 × 10^−4^). While results from pairwise comparisons are not significant after correcting for multiple tests, the comparisons with the lowest *p* values largely included prey items X8a, X16, X18, and X91 in them and so these prey items likely contributed to the significant result. When examining each site separately, sizes of individuals that consume different prey do not differ significantly (*p* values range from 0.088 to 0.59; Figure A1).

### 3.2 Population Genetic Analyses

We obtained *COI* sequences for 20 individuals from Huahine, 28 individuals from Rangiroa, 20 individuals from Makemo, and 23 individuals from Rurutu (GenBank accession numbers PV534881‐PV534971). We inferred alleles of *MIL2* for 18 individuals from Huahine, 27 individuals from Rangiroa, 21 individuals from Makemo, and 23 individuals from Rurutu. We inferred alleles of *MIL3* for 18 individuals from Huahine, 28 individuals from Rangiroa, 18 individuals from Makemo, and 24 individuals from Rurutu. For other individuals, we either did not obtain successful amplifications from their extracted genomic DNAs or could not determine *MIL2* and/or *MIL3* alleles from chromatograms (which in some cases appeared to include representatives of other O‐superfamily conotoxin loci). In few cases, we inferred new *MIL2* and *MIL3* alleles that differed from sequences of previously described alleles (Duda and Lee 2009b), including a sequence that differed at one site from allele “MIL2‐A1” that was observed from two individuals from Makemo, one individual from Rangiroa, and one individual from Huahine; a sequence that differed at two sites from allele “MIL2‐A1” that was observed from single individuals from Makemo and Rangiroa; a sequence that differed at one site from allele “MIL2‐A1” that was observed from one individual from Makemo; a sequence that differed at two sites from allele “MIL2‐A1” that was observed from one individual from Makemo; a sequence that differed at one site from allele “MIL3‐B” that was observed from an individual from Rurutu; and a sequence that differed at one site from allele “MIL3‐F1” that was observed from one individual from Huahine (GenBank accession numbers PV548805‐PV548810).

For all three loci, F_ST_ values calculated from pairwise comparisons among different sites in French Polynesia are small and not significant (i.e., from −0.024 to 0.023 for *COI*, from −0.016 to 0.023 for *MIL2*, and from −0.021 to 0.020 for *MIL3*) (Tables 3, 4, 5). F_ST_ values from analysis of *COI* sequences for comparisons that include sites in French Polynesia and Rapa Nui are, on the other hand, relatively large (i.e., range from 0.091 to 0.168) and significant and comparable to those estimated for the population pairs Rapa Nui‐Guam and Rapa Nui‐American Samoa (i.e., 0.121 and 0.143) (Table 3). Values from analysis of *COI* sequence for pairwise comparisons between populations from French Polynesia, Guam, and American Samoa are small and not significant (i.e., between −0.039 and 0.020), except for the comparison between Rangiroa and American Samoa which is relatively small (i.e., 0.026) but shows significance (i.e., *p* value = 0.035) (Table 3). F_ST_ values calculated for the *MIL2* and *MIL3* loci are large and significant for pairwise comparisons among populations from French Polynesia and Rapa Nui (i.e., between 0.463 and 0.626 for *MIL2* and between 0.021 and 0.118 for *MIL3*) (Tables 4 and 5). For *MIL2*, values are also significant for most pairwise comparisons involving sites in French Polynesia and Guam and American Samoa (except for the comparison involving Guam and Huahine), although they are smaller (i.e., from 0.057 to 0.191) than those from comparisons that include Rapa Nui (Table 4). For *MIL3*, most values are small (i.e., between 0.006 and 0.100) and not significant for comparisons involving sites in French Polynesia and Guam and American Samoa; the only exception is for the comparison involving Huahine and American Samoa (i.e., F_ST_ value = 0.101 and *p* value = 0.041) (Table 5).

**TABLE 3:** Patterns of differentiation at *COI* sequences.

	Guam	American Samoa	Huahine	Rangiroa	Rurutu	Makemo	Rapa Nui
Guam		0.831	0.842	0.518	0.776	0.617	**0.000**
American Samoa	−0.012		0.352	**0.035**	0.464	0.290	**0.000**
Huahine	−0.015	0.020		0.207	0.760	0.522	**0.000**
Rangiroa	−0.039	**0.026**	0.010		0.073	0.327	**0.000**
Rurutu	−0.012	−0.001	−0.011	0.023		0.967	**0.000**
Makemo	−0.009	0.005	−0.005	0.003	−0.024		**0.000**
Rapa Nui	**0.121**	**0.143**	**0.091**	**0.095**	**0.168**	**0.166**	

*Note:* F_ST_ values below the diagonal, *p* values above the diagonal. *p* values less than 0.05 and their corresponding F_ST_ values are in bold typeface.

**TABLE 4:** Patterns of differentiation at *MIL2* sequences.

	Guam	American Samoa	Huahine	Rangiroa	Rurutu	Makemo	Rapa Nui
Guam		0.469	0.168	**0.014**	**0.008**	**0.040**	**0.000**
American Samoa	−0.008		**0.027**	**0.000**	**0.001**	**0.006**	**0.000**
Huahine	0.062	**0.072**		0.139	0.321	0.717	**0.000**
Rangiroa	**0.139**	**0.191**	0.023		0.618	0.293	**0.000**
Rurutu	**0.110**	**0.165**	−0.000	−0.013		0.481	**0.000**
Makemo	**0.057**	**0.099**	−0.019	0.002	−0.010		**0.000**
Rapa Nui	**0.731**	**0.767**	**0.626**	**0.463**	**0.523**	**0.580**	

*Note:* F_ST_ values below diagonal, *p* values above diagonal. *p* values less than 0.05 and their corresponding F_ST_ values are in bold typeface.

**TABLE 5:** Patterns of differentiation at *MIL3* sequences.

	Guam	American Samoa	Huahine	Rangiroa	Rurutu	Makemo	Rapa Nui
Guam		0.819	0.032	0.203	0.080	0.284	**0.000**
American Samoa	−0.024		**0.041**	0.152	0.056	0.251	**0.000**
Huahine	0.100	**0.101**		0.250	0.525	0.522	**0.017**
Rangiroa	0.010	0.017	0.020		0.500	0.926	**0.000**
Rurutu	0.032	0.041	−0.003	−0.009		0.794	**0.000**
Makemo	0.006	0.009	0.008	−0.021	−0.017		**0.000**
Rapa Nui	**0.236**	**0.235**	**0.021**	**0.134**	**0.100**	**0.118**	

*Note:* F_ST_ values below diagonal, *p* values above diagonal. *p* values less than 0.05 and their corresponding F_ST_ values are in bold typeface.

**FIGURE 6:**
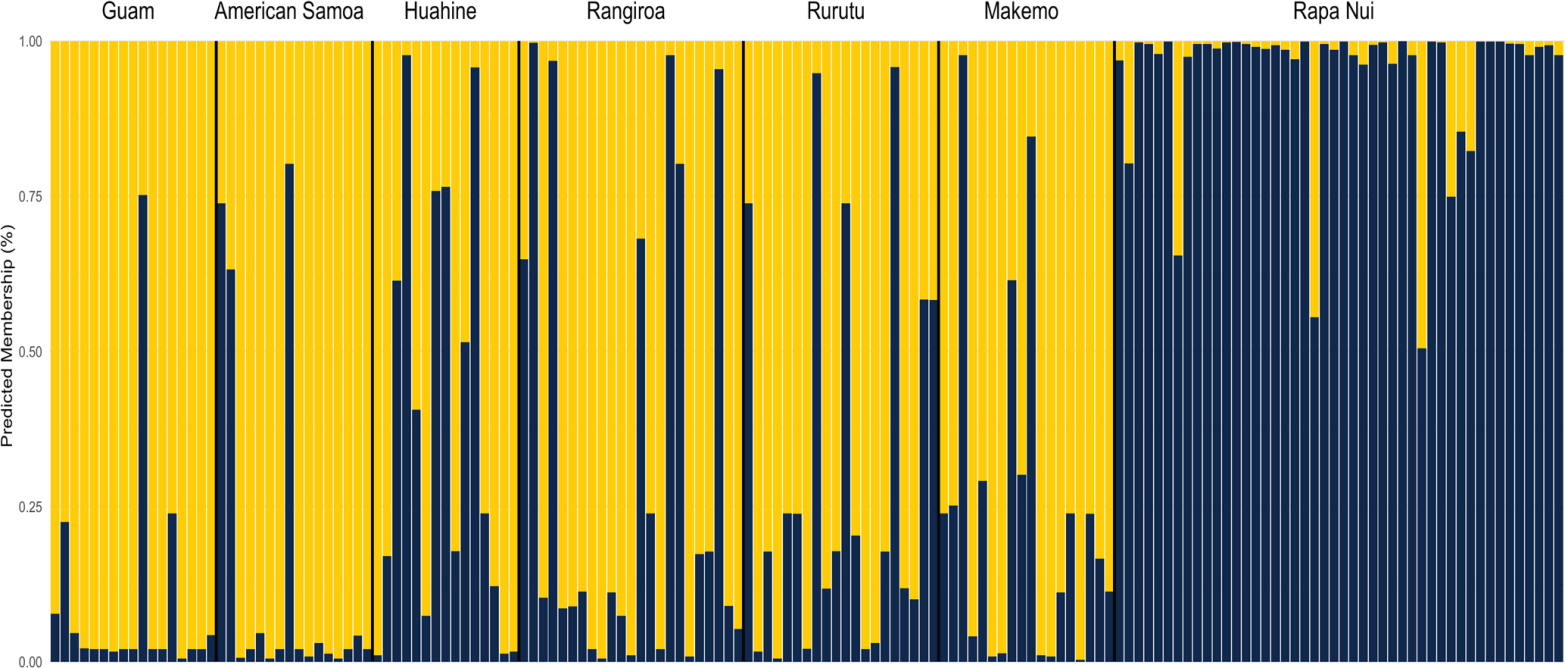
Predicted assignments to clusters for *K* = 2 as determined with output from Structure and no admixture model for analyses of *MIL2* and *MIL3*.

**FIGURE A2:**
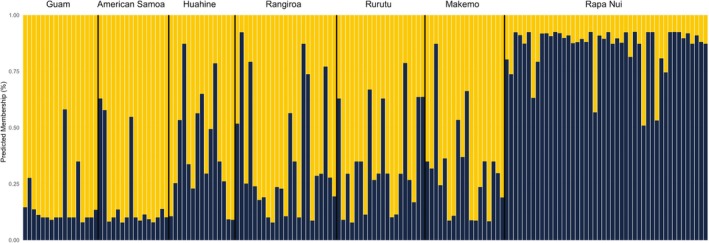
Predicted membership assignments to clusters for *K* = 2 as determined with output from Structure with admixture model for analyses of *MIL2* and *MIL3*.

**FIGURE A3:**
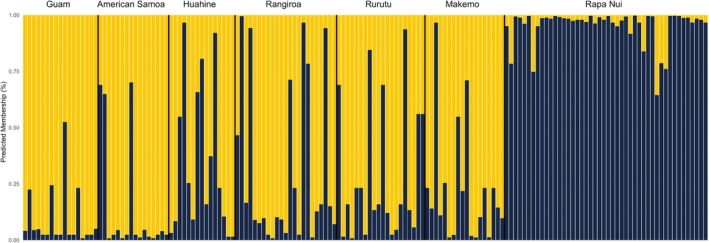
Predicted assignments to clusters for *K* = 2 as determined with output from rmaverick with no admixture model for analyses of *MIL2* and *MIL3*.

**FIGURE A4:**
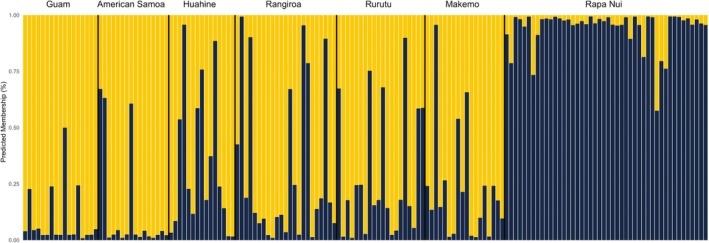
Predicted assignments to clusters for *K* = 2 as determined with output from rmaverick with no admixture model for analyses of *MIL2* and *MIL3*.

To further investigate the structure of populations of *C. miliaris* , we examined genotypes of individuals at the *MIL2* and *MIL3* loci to evaluate support for different numbers of population clusters (*K* values) and the assignment of individuals to these clusters with Structure and rmaverick based on models of admixture and no admixture. Both approaches and both models support a *K* of 2, while results from both approaches support models of no admixture (Tables A1, A2, A3). Assignment plots from different analyses and models are similar in that most individuals from Guam and American Samoa are assigned to one cluster and most individuals from Rapa Nui are assigned to the other cluster with high support (Figure 6; Figures A2, A3, A4). Individuals from sites in French Polynesia show more individuals with a higher assignment percentage to this latter cluster than do individuals from Guam and American Samoa (Figure 6). Although average assignment percentages to one cluster for individuals from Guam and American Samoa are greater than 80.9%, those from sites in French Polynesia range between 60.6% and 77.4%, while those at Rapa Nui range between 4.9% and 14.2% (depending on the approach and model used; see Table A4).

## 4 Discussion

Populations at sites in French Polynesia exhibit overlap in prey utilization. Moreover, while diets at most sites in French Polynesia are significantly different from diets at Guam and American Samoa, prey items of populations at several sites show overlap with those at Rapa Nui. Populations at several sites in French Polynesia also show broad dietary breadths with diversity metric values in some cases greater than those determined for the population at Rapa Nui. Although we found a significant association between sizes of individuals and the prey they utilize, differences in diet among populations do not appear to reflect ontogenetic shifts in diet and differences in sampled size distributions of populations. In addition, although analysis of *COI* sequences shows that populations from French Polynesia are closely allied with populations at Guam and American Samoa and genetically differentiated from the population at Rapa Nui, analyses of two venom‐associated genes reveal that populations at French Polynesia exhibit greater levels of admixture with the population at Rapa Nui than populations from Guam and American Samoa.

### 4.1 Patterns of Variation in Diet

We aimed to evaluate patterns of variation in feeding ecology of *C. miliaris* at a finer spatial scale than previously examined to determine if they differ across smaller spatial scales to the same extent that they do across larger scales (Weese and Duda 2019). Our results reveal that populations from geographically proximate locations (e.g., within French Polynesia) tend to exhibit greater similarity in diet than distant ones. Indeed, the largest estimates of dietary overlap include values of all comparisons among populations at sites in French Polynesia; the smallest values occur for most of the comparisons among populations from distant locations.

The population at American Samoa, which is closest geographically to sites sampled in French Polynesia, shows very little overlap in diet with populations in French Polynesia, with only one prey item shared at American Samoa and Rurutu (which is also shared with populations at Guam and Rapa Nui; see below). Because the individuals from which prey items were determined at American Samoa are larger in size than those sampled at most sites elsewhere, the lack of overlap could reflect size‐specific differences in the diets of *C. miliaris* (see below regarding possible ontogenetic shifts in diet). Nonetheless, individuals examined at the lagoon site at Rangiroa were also comparatively large‐sized but do not share any of the same prey items that were detected at American Samoa. Moreover, the prey item that is shared among populations at American Samoa, Rurutu, Guam, and Rapa Nui is consumed by relatively small‐sized individuals at these three latter sites. Interestingly, the populations sampled at Guam and the lagoon site at Rangiroa exhibit the largest dietary overlap in which most individuals at these sites share the same prey item which also represents a relatively rare prey item that is only rarely consumed by individuals at Rurutu (i.e., identified from just two of 26 individuals).

While dietary overlap of populations at sites at Guam and American Samoa and those from most sites at French Polynesia and Rapa Nui is low, several populations at sites in French Polynesia and Rapa Nui share similar prey items. These include the populations at Huahine, Makemo, and Rurutu in French Polynesia. On the contrary, the populations at the two sites at Rangiroa share no prey items in common with the population at Rapa Nui. Moreover, while measures of dietary breadth of populations at Huahine and the lagoon site at Rangiroa are relatively low and comparable to those of populations at Guam and American Samoa, those from populations at Rurutu, Makemo, and the seaward site at Rangiroa are more similar to (and in some cases greater than) values estimated for the population at Rapa Nui. Indeed, populations from these three sites are the only ones outside of Rapa Nui to include prey items other than eunicids. This result is especially surprising given the hypothesis that the population at Rapa Nui exhibits a broad dietary breadth due to ecological release as a result of the absence of congeners at this site (Kohn 1978). At sites in French Polynesia, *C. miliaris* co‐occurs with many congeners and so ecological release does not appear to be a reasonable explanation for the increased dietary breadth for populations here. A potential explanation for the broad diet of *C. miliaris* at sites in French Polynesia (as well as at Rapa Nui) is that the region holds a greater diversity of prey for vermivorous cone snails than at Guam and American Samoa.

Although populations at sites in French Polynesia show overlap in diet, they also exhibit, as described above, differences in dietary breadth. For example, we only detected prey items other than eunicids from sites at Rurutu, Makemo, and the seaward site at Rangiroa. If populations from geographically proximate sites tend to share similar diets, what contributes to the differences in dietary breadth among these populations? A possible explanation is that sample sizes of certain populations were inadequate for assessing dietary breadth. Indeed, the populations from sites at French Polynesia with relatively narrow dietary breadths, Huahine and the lagoon site at Rangiroa, also had the smallest sample sizes (eight and six, respectively) compared to populations at sites elsewhere (which ranged from 11 to 26). Therefore, increased sampling from sites in French Polynesia is needed to determine if and how dietary breadth varies among nearby locations.

### 4.2 Ontogenetic Shifts in Diet

The relationship between shell size and prey utilization suggests that some of the variation in diet detected for particular populations reflects differences in diets among different size classes (i.e., changes in diet during ontogeny). For example, several prey items were only identified from feces of relatively small‐sized individuals (i.e., eunicids X9, X21, X22, X90, and X91, and onuphid O2; Figure 4) or relatively large‐sized individuals (i.e., eunicids X16 and X18; Figure 4). In addition, although size distributions of individuals at most sites are largely overlapping, some sites include an excess of small (i.e., Makemo) or large‐sized individuals (e.g., the lagoon at Rangiroa and American Samoa) which is likely due to recent recruitment or the lack thereof at these sites (Duda and Vergara‐Florez 2025). The most commonly consumed prey item at the lagoon site at Rangiroa (i.e., X6) and one of the prey items consumed by relatively small‐sized individuals at Makemo (i.e., X9) are also consumed by smaller or larger‐sized individuals at other sites (Figure 4). However, two of the prey items at Guam (i.e., X16 and X18) and two of the prey items at Makemo (i.e., X22 and X90) were exclusively found only in relatively large and small‐sized individuals, respectively, at these sites and so may represent prey items that are utilized by individuals of different size classes.

Some of the prey items are either exclusively (i.e., X16 and X18) consumed by or represent the most common prey item (i.e., X8a) of relatively large‐sized individuals at American Samoa (Figures 2 and 4). This suggests that the observed significant differences in diet between American Samoa and other sites reflect differences in sizes of specimens that were compared from American Samoa and elsewhere and that *C. miliaris* undergoes ontogenetic shifts in diet. Nonetheless, large‐sized individuals at other sites do not appear to specialize in the same prey items that are consumed by similarly sized individuals at American Samoa (see Figure 4) and so the differences in diets we observed among sites do not appear to be driven by differences in the size frequency distribution of individuals from different sites. While previous work has shown that diets of other *Conus* species change during ontogeny (Chang and Duda 2016; Nybakken and Perron 1988; Rogalski et al. 2023), examination of diets of smaller individuals of *C. miliaris* at American Samoa or broader size distributions of it elsewhere is needed to determine if it also exhibits ontogenetic shifts in diet.

### 4.3 Population Genetic Patterns

In agreement with past investigations of the genetic population structure of *C. miliaris* in the Indo‐West Pacific (Duda and Lee 2009b), populations at sites in French Polynesia are not differentiated from populations in Guam and American Samoa and show strong differentiation from the population in Rapa Nui based on analysis of *COI* sequences. Indeed, while F_ST_ values calculated among populations from Guam, American Samoa, and French Polynesia are relatively low, those calculated for comparisons involving Rapa Nui are much larger (Table 3). These results support previous interpretations that the geographic isolation of Rapa Nui and relative lack thereof at other locations in the Indo‐West Pacific contribute to the pattern of structure observed (Duda and Lee 2009b).

In contrast to results from the analysis of *COI* sequences, analyses of sequences of the two conotoxin loci reveal a different pattern. In particular, although populations from sites in French Polynesia generally are not strongly differentiated from populations in Guam and American Samoa, estimated levels of genetic differentiation (i.e., F_ST_ values) between populations in French Polynesia and Rapa Nui are lower than those for comparisons involving populations in Rapa Nui and in Guam and American Samoa (Tables 4 and 5). Moreover, the structure analyses performed support the existence of two population clusters in which most individuals from Guam and American Samoa are assigned to one cluster and those from Rapa Nui are assigned to the other. Although some individuals from sites in French Polynesia show high levels of predicted membership to the former cluster, many individuals from these sites also exhibit membership to the latter one (Figure 6). Hence, the population of *C. miliaris* from French Polynesia appears to be admixed and comprised of individuals with a greater frequency of genotypes (and alleles) as observed at Rapa Nui than do populations from Guam and American Samoa.

Several factors may contribute to the different patterns of genetic population structure that were detected for the sequences of *COI* and the two conotoxin loci. Previous estimates of gene flow from analyses of *COI* sequences suggest that rates of migration are greater from Rapa Nui toward the rest of the Indo‐West Pacific as opposed to toward Rapa Nui (Duda and Lee 2009b). The patterns of variation at the two conotoxin loci may also reflect gene flow from Rapa Nui to the most geographically proximate location for which we currently have data (i.e., French Polynesia).

If migration from Rapa Nui to French Polynesia contributes to admixture of the latter population, why is the pattern different for mitochondrial (i.e., *COI*) and nuclear (i.e., conotoxin) loci with an apparent higher rate of admixture of the latter? One potential though seemingly unlikely explanation is that there is sex‐biased dispersal in which gene flow from Rapa Nui is only achieved via migration of males (we assume that mitochondria of cone snails are maternally transmitted as they are in many animals). Nonetheless, we cannot envision how sex‐biased dispersal might occur given that migration is likely accomplished through dispersal and settlement of larvae which likely include an even mix of individuals that will develop into either males or females. Another explanation may be related to differences in effective population sizes (and the assumption that the population at Rapa Nui has a small one) and selection against deleterious mitochondrial genomes. Populations with small effective population sizes are prone to accumulating deleterious mutations, especially for nonrecombining genomes like those of mitochondria (Gabriel et al. 1993). For example, *Drosophila* species exhibit biased rates of introgression of mitochondrial genomes in which the genome of a species with a small effective population size has been replaced by that of one with a larger effective population size via past hybridization events (Llopart et al. 2014). Hence, we hypothesize that mitochondrial genomes of the Rapa Nui population are deleterious compared to those of populations elsewhere (owing to the difference in effective population sizes) and selection reduces the frequencies of these genomes when migration occurs from Rapa Nui to other locations. Finally, the apparent greater rate of admixture of nuclear alleles from Rapa Nui to populations in French Polynesia may be due to their selective advantage. Indeed, conotoxin loci are functional loci that are expressed in the venoms of cone snails and primarily utilized for prey capture. Acquisition of novel conotoxin alleles may facilitate access to previously unexploited prey items or provide the opportunity to overcome evolved resistance of current ones. We plan to test these latter two hypotheses by comparing mitochondrial genomes and examining rates of admixture of additional nuclear loci of populations from Rapa Nui and elsewhere.

### 4.4 Caveats

Sample sizes of several of the populations from French Polynesia (in terms of dietary analyses) are smaller than those from populations elsewhere (Table 1) and this reduces our power for detecting significant differences in diet among them. In addition, while habitat type is known to affect access to and availability of prey items of cone snails (Kohn 1959), the habitats from locations where diets have been characterized differ and in some cases differ considerably. For example, the lagoon habitat at Rangiroa is quite distinct from habitats at other sites in that it represents a subtidal reef with little wave energy, while other sites typically are comprised of reef flats near fringing reefs with sometimes considerable wave energy. Nonetheless, despite the small sample sizes and different habitat types, populations at sites in French Polynesia share similar prey items. In addition, diets were characterized at different points in time. This may account for observed differences in diets if diets of populations are temporally dynamic. Nonetheless, given unpublished results for populations of cone snail species that have been surveyed at a few sites at different times and comparison of dietary characterizations of *C. miliaris* at Rapa Nui that were conducted across different decades (i.e., Kohn (1978) and Weese and Duda (2019)), we feel that this is unlikely to explain the geographic patterns of dietary variation that we report herein. Moreover, we did not perform any assessments of prey availability and so do not know if differences in prey abundance among sites or habitat types contributed to observed differences in diet, although several of the prey of *C. miliaris* must be broadly distributed as we obtained identical or similar sequences of prey items from disparate locations (see Figure 2).

## 5 Conclusion

Diets of *C. miliaris* show regional patterns of differentiation with largely little to no overlap among widely separated populations and greater overlap among nearby sites within regions. Nonetheless, although the diet of *C. miliaris* at Rapa Nui at the extreme southeastern edge of the range of this species was previously considered to be distinct in terms of its composition of prey items and breadth, diets of populations from French Polynesia exhibit overlap with the diet of the Rapa Nui population and remarkably exhibit similar levels of breadth to it. While ecological release resulting from the absence of competitors (i.e., congeners) at Rapa Nui may still account for its broad diet (Kohn 1978), this explanation does not seem sufficient to explain the broad diet at French Polynesia given the presence of congeners. Instead, as suggested by the greater levels of admixture between Rapa Nui and French Polynesia (than between Rapa Nui and Guam and American Samoa), the broad diet of these populations may reflect the migration of this ecological phenotype from Rapa Nui. Here, we posit that this phenotype has a genetic basis (that may be partially determined from chemosensory gene repertoires that enable detection of prey), arose within the population at Rapa Nui owing to ecological release and the relative isolation of this population, and then dispersed into French Polynesia (and potentially other nearby locations such as the Gambier Islands and Pitcairn Islands group which are part of the Tuamotu Archipelago and occur further to the southeast/east from French Polynesia) via migration, possibly via the westward flow of the South Equatorial Current (Rougerie and Rancher 1994). Alternatively, the region of the Indo‐West Pacific where these populations occur may share biotic and/or abiotic conditions that provide similar communities of and access to potential prey that are responsible for their overlapping and broad diets. We plan to address these hypotheses through comparisons of diets of other cone snail species and population genomic analyses of *C. miliaris* .

## Author Contributions

**Thomas F. Duda Jr:** conceptualization (lead), formal analysis (lead), funding acquisition (lead), investigation (lead), methodology (lead), project administration (lead), resources (lead), software (lead), supervision (lead), visualization (lead), writing – original draft (lead), writing – review and editing (equal). **Diana Carolina Vergara‐Flórez:** investigation (supporting), writing – review and editing (equal).

## Conflicts of Interest

The authors declare no conflicts of interest.

## Data Availability

The data that support the findings of this study are openly available in GenBank at https://www.ncbi.nlm.nih.gov/nucleotide/, reference number PV540063‐PV540125, PV534881‐PV534971, PV548805‐PV548810.

## Appendix A

**TABLE A1:** Results from Structure/Structure Harvester with no admixture model using the Evanno method.

*K*	Mean LnP(*K*)	SD LnP(*K*)	Ln′(*K*)	|Ln″(*K*)|	Delta *K*
1	−1034.79	0.17	NA	NA	NA
**2**	**−934.52**	**0.12**	**100.27**	**125.86**	**1023.86**
3	−960.11	2.43	−25.59	47.97	19.76
4	−937.73	2.74	22.38	51.70	18.88
5	−967.05	9.74	−29.32	23.88	2.45
6	−972.49	10.30	−5.44	9.68	0.94
7	−987.61	8.32	−15.12	NA	NA

*Note:* Means and standard deviations (SD) of estimated log likelihood of probability of *K* (LnP(*K*)) are reported as well as first derivatives (Ln′(*K*)) and absolute value of second derivates (|Ln″(*K*)|) of the likelihood score, and Delta *K* statistics for different *K* values. The row that includes the *K* value with greatest support is given in bold typeface.

**TABLE A2:** Results from Structure/Structure Harvester with admixture model using the Evanno method.

*K*	Mean LnP(*K*)	Stdev LnP(*K*)	Ln′(*K*)	|Ln″(*K*)|	Delta *K*
1	−1034.72	0.09	NA	NA	NA
**2**	**−958.73**	**5.72**	**75.99**	**146.25**	**25.58**
3	−1028.99	6.20	−70.26	127.96	20.63
4	−971.29	4.16	57.70	74.08	17.82
5	−987.67	6.48	−16.38	23.69	3.66
6	−980.36	6.42	7.31	65.64	10.22
7	−1038.69	19.49	−58.33	NA	NA

*Note:* Means and standard deviations (SD) of estimated log likelihood of probability of *K* (LnP(*K*)) are reported as well as first derivatives (Ln′(*K*)) and absolute value of second derivates (|Ln″(*K*)|) of the likelihood score, and Delta *K* statistics for different *K* values. The row that includes the *K* value with greatest support is given in bold typeface.

**TABLE A3:** Results from rmaverick.

*K*	GTI, no admixture (SD)	GTI, admixture (SD)
1	−1072 (0.000)	−1072 (0.000)
**2**	**−1053 (0.028)**	**−1054 (0.043)**
3	−1065 (0.033)	−1066 (0.061)
4	−1081 (0.032)	−1083 (0.054)
5	−1096 (0.032)	−1099 (0.052)
6	−1111 (0.030)	−1114 (0.055)
7	−1125 (0.028)	−1128 (0.047)

*Note:* Support for different values of *K* given as generalized thermodynamic integration log evidence scores (GTI) for models with no admixture and admixture and their standard deviations (SD) are provided. The row that includes the *K* value with greatest support is given in bold typeface.

**TABLE A4:** Average assignment percentages to cluster 1 for each site based on results from Structure and rmaverick using models of no admixture and admixture for *K* = 2.

Site	Structure, no admixture	Structure, admixture	rmaverick, admixture	rmaverick, no admixture
Guam	0.907	0.841	0.906	0.907
American Samoa	0.848	0.809	0.854	0.862
Huahine	0.612	0.606	0.650	0.657
Rangiroa	0.678	0.636	0.684	0.686
Rurutu	0.690	0.647	0.711	0.709
Makemo	0.752	0.690	0.774	0.772
Rapa Nui	0.054	0.142	0.049	0.058
